# Emodin Suppresses Migration and Invasion through the Modulation of CXCR4 Expression in an Orthotopic Model of Human Hepatocellular Carcinoma

**DOI:** 10.1371/journal.pone.0057015

**Published:** 2013-03-05

**Authors:** Kanjoormana Aryan Manu, Muthu K. Shanmugam, Tina H. Ong, Aruljothi Subramaniam, Kodappully Sivaraman Siveen, Ekambaram Perumal, Ramar Perumal Samy, Pradeep Bist, Lina H. K. Lim, Alan Prem Kumar, Kam M. Hui, Gautam Sethi

**Affiliations:** 1 Department of Pharmacology, Yong Loo Lin School of Medicine, National University of Singapore, Singapore, Singapore; 2 Division of Cellular and Molecular Research, Humphrey Oei Institute of Cancer Research, National Cancer Centre, Singapore, Singapore; 3 Molecular Toxicology Lab, Department of Biotechnology, Bharathiar University, Coimbatore, Tamil Nadu, India; 4 Infectious Diseases Programme, Department of Microbiology, National University of Singapore, Singapore, Singapore; 5 Department of Physiology, National University of Singapore, Singapore, Singapore; 6 Immunology Program, National University of Singapore, Singapore, Singapore; 7 Cancer Science Institute of Singapore, National University of Singapore, Centre for Translational Medicine, Singapore, Singapore; 8 School of Biomedical Sciences, Faculty of Health Sciences, Curtin University, Perth, Western Australia; 9 Department of Biological Sciences, University of North Texas, Denton, Texas, United States of America; The University of Hong Kong, Hong Kong

## Abstract

Accumulating evidence(s) indicate that CXCL12-CXCR4 signaling cascade plays an important role in the process of invasion and metastasis that accounts for more than 80% of deaths in hepatocellular carcinoma (HCC) patients. Thus, identification of novel agents that can downregulate CXCR4 expression and its associated functions have a great potential in the treatment of metastatic HCC. In the present report, we investigated an anthraquinone derivative, emodin for its ability to affect CXCR4 expression as well as function in HCC cells. We observed that emodin downregulated the expression of CXCR4 in a dose-and time-dependent manner in HCC cells. Treatment with pharmacological proteasome and lysosomal inhibitors did not have substantial effect on emodin-induced decrease in CXCR4 expression. When investigated for the molecular mechanism(s), it was observed that the suppression of CXCR4 expression was due to downregulation of mRNA expression, inhibition of NF-κB activation, and abrogation of chromatin immunoprecipitation activity. Inhibition of CXCR4 expression by emodin further correlated with the suppression of CXCL12-induced migration and invasion in HCC cell lines. In addition, emodin treatment significantly suppressed metastasis to the lungs in an orthotopic HCC mice model and CXCR4 expression in tumor tissues. Overall, our results show that emodin exerts its anti-metastatic effect through the downregulation of CXCR4 expression and thus has the potential for the treatment of HCC.

## Introduction

Hepatocellular carcinoma (HCC) is a highly aggressive and deadly malignancy representing the fifth most common cancer worldwide and the fourth leading cause of cancer related deaths worldwide [1], [2]. Although surgical resection and use of conventional chemotherapy have slightly improved the outcome in HCC patients of late, the long-term prognosis remains unsatisfactory because of its inherent capacity of invasiveness and metastasis. Chemokine receptors have been reported to be involved in various aspects of HCC initiation and progression, specifically in migration, invasion and metastasis [3], [4]. Chemokines are a superfamily of small secreted molecules that consists of 40 ligands and at least 20 corresponding receptors [5]. Based on the position of the first two conserved cysteine residues, the chemokines can be separated into four categories, CXC, CC, C, and CX3C, and exert their biological effects through selective membrane-bound G protein-coupled receptors [4], [5]. Among the large family of chemokines and their receptors, the most extensively studied is CXCR4/CXCL12 signaling cascade, which is expressed by various types of tumor cells, including, liver [6]–[9] and plays a critical role in the inflammatory responses, angiogenesis, tumor growth, invasion, and metastasis [10]–[12].

The effects of the CXCR4/CXCL12 axis on HCC are considered to be multidimensional and it has been implicated in both the homing of tumor cells to specific organs, as well as the growth of tumor cells at specific locations, which are most likely mediated by the effects of CXCR4 on migration, invasion, and metastasis [7]–[9]. For example, Xiang et al., recently showed that CXCR4 expression significantly correlated and was predictive of bone metastasis in HCC patients and also decreased overall median survival [8]. Also it has been found that the nuclear accumulation of CXCR4 is associated with a higher risk of lymph node metastasis in HCC [13]. Thus, CXCR4 may not only prove useful for predicting distant organ metastasis, but may also serve as a therapeutic target for HCC.

In the present report, we investigated the effect of emodin (1, 3, 8-trihydroxy-6methylanthraquinone), an active component found in the root and rhizome of *Rheum palmatum L*. (Polygonaceae) as a novel regulator of CXCR4 expression and function in HCC. Although emodin has been previously reported to possess anti-inflammatory, anti-proliferative, pro-apoptotic and chemopreventive activities in many tumor cell lines and animal models [14]–[18], whether emodin can modulate invasion and metastasis in HCC has not been investigated before. Also, our group is currently investigating the detailed anticancer mechanisms of emodin in HCC and has observed that it can cause suppression of signal transducer and activator of transcription 3 signaling cascade and enhancement of TNF-related apoptosis-inducing ligand-induced apoptosis in HCC cells (manuscripts communicated). The present findings clearly indicate that emodin can downregulate constitutive CXCR4 expression in HCC cells and tissues derived from orthotopic mice model. This inhibition occurred at the transcriptional level and contributed to the suppression of metastasis in an orthotopic HCC mice model.

## Results

**Figure 1:**
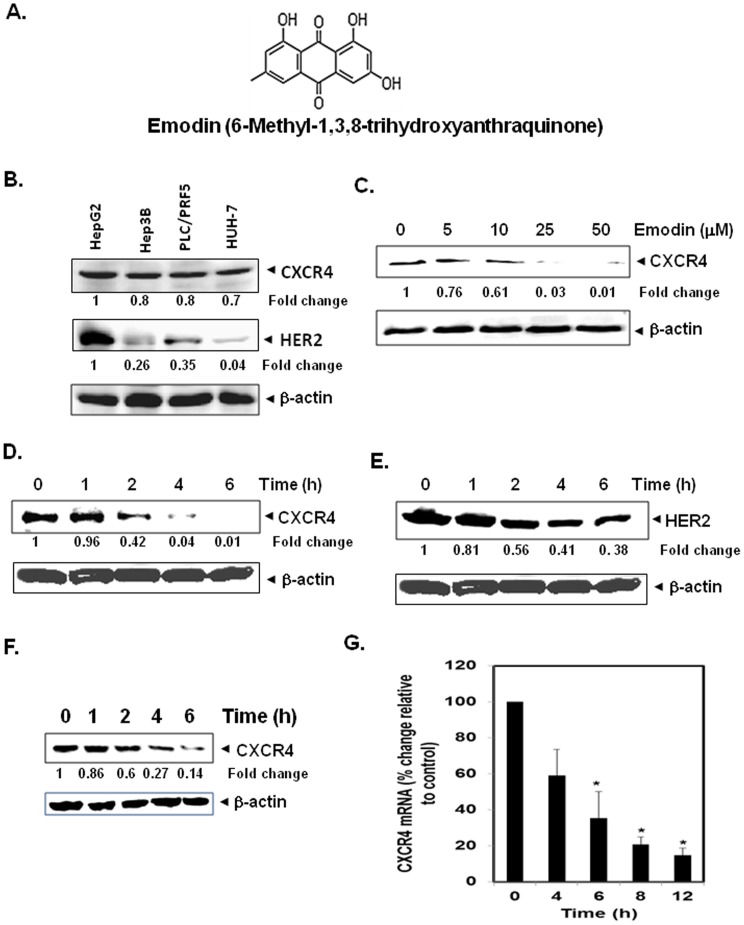
Emodin suppresses CXCR4 in HCC cells. A, The chemical structure of emodin. B, Western blot analysis of CXCR4 and HER2 expression in HCC cells. Whole-cell extracts of HepG2, Hep3B, HUH7, and PLC/PRF5 (30 µg) were resolved on SDS-PAGE gel and probed with anti-CXCR4 and HER2 antibodies. As a loading control, stripped membrane was probed with β-actin antibody. C, Emodin suppresses CXCR4 levels in a dose-dependent manner. HepG2 cells (1×10^6^) were treated with the indicated concentrations of emodin for 6 h. Whole cell extracts were then prepared, and 30 µg of protein was resolved on SDS-PAGE, electrotransferred onto nitrocellulose membranes, and probed for CXCR4. The same membrane was stripped and reprobed with β-actin antibody to show equal protein loading. D, Emodin suppresses CXCR4 levels in a time-dependent manner. HepG2 cells (1×10^6^) were treated with 50 µM emodin for the indicated times, after which Western blotting was done as described above. E, Effect of emodin on HER2 expression in HepG2 cells. HepG2 cells (1×10^6^) were treated with 50 µM emodin for the indicated times, after which Western blotting for HER2 was done as described above. The same membrane was stripped and reprobed with β-actin antibody to show equal protein loading. The representative results of two independent experiments are shown. F, Emodin suppresses expression of CXCR4 protein expression in Hep3B cells. Cells were incubated with 50 µM emodin for indicated times. Whole-cell extracts were prepared and analyzed by Western blot analysis using antibodies against CXCR4. The same membrane was stripped and reprobed with β-actin antibody to show equal protein loading. Representative results of two independent experiments are shown. G, Emodin suppresses expression of CXCR4 mRNA expression in Hep3B cells. Hep3B cells were treated with 50 µM emodin for indicated times. Total RNA was isolated and analyzed by quantitative real time PCR assay as described in Materials and Methods. GAPDH was shown to equal loading of total RNA. Representative results of two independent experiments are shown.

The present study was designed to investigate the effect of emodin (with structure shown in Fig. 1A) on both CXCR4 expression and function in HCC cells and orthotopic mice model.

### Emodin Suppresses the Expression of CXCR4 Protein in HCC Cells

Several lines of evidence implicate the role of CXCR4 in both invasion and metastasis in HCC [1], [6], [7], [13]. Hence, we first investigated the expression level of CXCR4 in four different HCC cell lines, namely HepG2, Hep3B, PLC/PRF5 and HUH7. As shown in Fig. 1B, it is clearly evident that CXCR4 is substantially expressed in all four different HCC cell lines. Because, HER2 has been shown to induce the expression of CXCR4 in tumor cells [19], we also analyzed the expression levels of HER2 in these four HCC cells. However, we found that there is no obvious relationship between CXCR4 and HER2 expression levels in HCC cell lines, as HER2 expression level was relatively lower in Hep3B and PLC/PRF5 cells that showed relatively high expression levels of CXCR4 (Fig. 1B). Interestingly, only in HepG2 cells, the expression level of HER2 was quite high and comparable to CXCR4 levels (Fig.1B). Hence, we first decided to investigate the effect of emodin on CXCR4 expression in detail in HepG2 cells. When HepG2 cells were incubated either with different concentrations of emodin for 6 h or with 50 µM of emodin for different times, emodin suppressed the expression of CXCR4 in a dose- (Fig. 1C) and time- (Fig. 1D) dependent manner. The exposure of cells to 50 µM emodin for 6 h significantly inhibited the CXCR4 expression as evident by time kinetics study in Fig. 1D. This downregulation was not due to decrease in cell viability as approximately 90% of cells were viable under these conditions (data not shown).

Since HER2 has been reported to regulate the expression of CXCR4 by stimulating CXCR4 translation and attenuating CXCR4 degradation [19], we also examined whether emodin affects CXCR4 expression through the modulation of HER2 expression in HCC cells. For this, HER2-overexpressing HepG2 cells were incubated with different concentrations of emodin for 6 h and then examined for HER2 expression by Western blot analysis using specific antibodies. We found that HER2 expression was partially affected after emodin treatment in HepG2cells (Fig. 1E), thus suggesting that downregulation of CXCR4 expression by emodin may not be completely due to modulation of HER2 expression. We further found that emodin also downregulated the expression of both protein (Fig. 1F) and mRNA (Fig. 1G) for CXCR4 in a time dependent manner in Hep3B cells, thereby suggesting that the effect of emodin on CXCR4 expression is not limited to a single HCC cell line.

### Downregulation of CXCR4 Expression by Emodin is not Mediated through its Degradation

**Figure 2:**
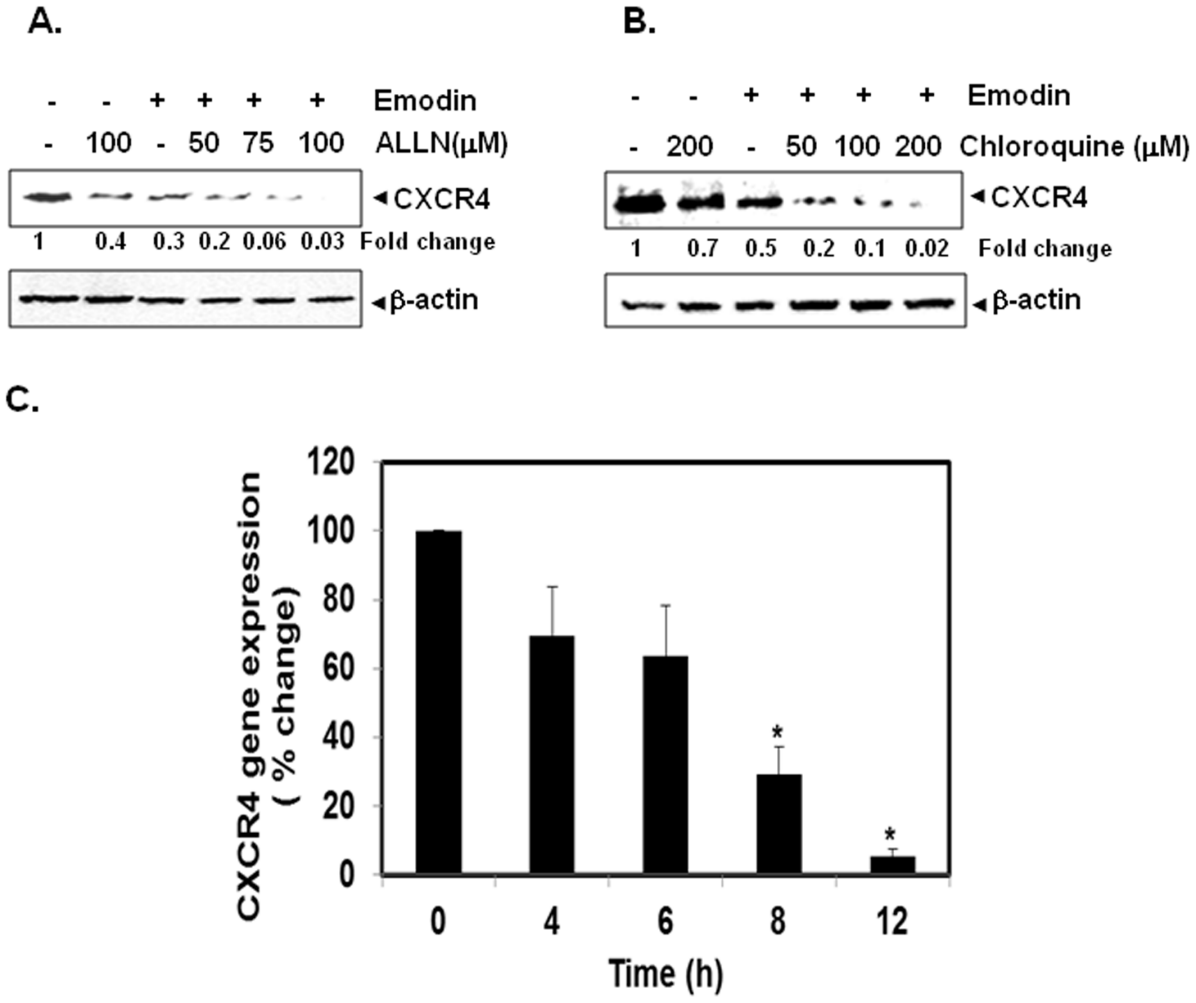
Emodin suppresses CXCR4 mRNA level in HCC cells. A and B, Emodin suppresses CXCR4, neither by proteasomal nor by lysosomal degradation. HepG2 cells (1×10^6^) were treated with indicated concentrations of ALLN or chloroquine for 1 h at 37°C, followed by treatment of 50 µM emodin for 6 h. Whole-cell extracts were prepared and analyzed by Western blot analysis using antibodies against CXCR4. The same blots were stripped and reprobed with β-actin antibody to show equal protein loading. Representative results of two independent experiments are shown. C, Emodin suppresses the expression of CXCR4 mRNA in HepG2 cells. HepG2 cells were treated with 50µM emodin for the indicated time intervals, after which cells were harvested after treatment and total RNA samples were extracted. 1µg portions of the respective RNA extracts then proceed for Reverse Transcription to generate corresponding cDNA. Real time PCR was performed to measure the relative quantities of CXCR4 mRNA using targeted TaqMan probes, with GAPDH as endogenous control for measurement of equal loading of RNA samples. Results were analyzed using Sequence Detection Software version 1.3 provided by Applied Biosystems. Relative gene expression was obtained after normalization with endogenous GAPDH and determination of the difference in threshold cycle (Ct) between treated and untreated cells using 2-ΔΔCt method.

Bhandari et al., 2007 have reported previously that CXCR4 undergoes ubiquitination at its lysine residue followed by degradation [20], therefore we next investigated the possibility that emodin may enhance the rate of CXCR4 degradation via the activation of proteasomes. To investigate this, we examined the ability of ALLN, a proteasome inhibitor, to block emodin -induced degradation of CXCR4. HepG2 cells were pretreated with ALLN for 1 h before being treated with emodin. As shown in Fig. 2A, ALLN had minimal effect on emodin-induced degradation of CXCR4, suggesting that this is an unlikely basis for the suppression of CXCR4 expression by emodin. Since CXCR4 has also been shown to undergo ligand-dependent lysosomal degradation [20], we next investigated the ability of chloroquine, a lysosomal inhibitor, to block emodin induced degradation of CXCR4. The cells were pretreated with chloroquine for 1 h before exposure to emodin. Our results showed that chloroquine at 200 µM only slightly prevented the degradation of CXCR4 (Fig. 2B), suggesting that this was arguably not the primary pathway for modulation of CXCR4 expression.

### Downregulation of CXCR4 by Emodin Occurs at the Transcriptional Level

Since emodin did not downregulate CXCR4 expression by enhancing its degradation in HepG2 cells, we next investigated whether suppression occurs at the transcriptional level instead. Cells were treated with emodin for different times and mRNA was extracted for quantitative real-time PCR analysis. As shown in Fig. 2C, emodin induced downregulation of CXCR4 mRNA expression in a time dependent manner, with significant reduction observed as early as 6 h after exposure.

### Emodin Suppresses NF-κB Activation in HCC Cells

**Figure 3:**
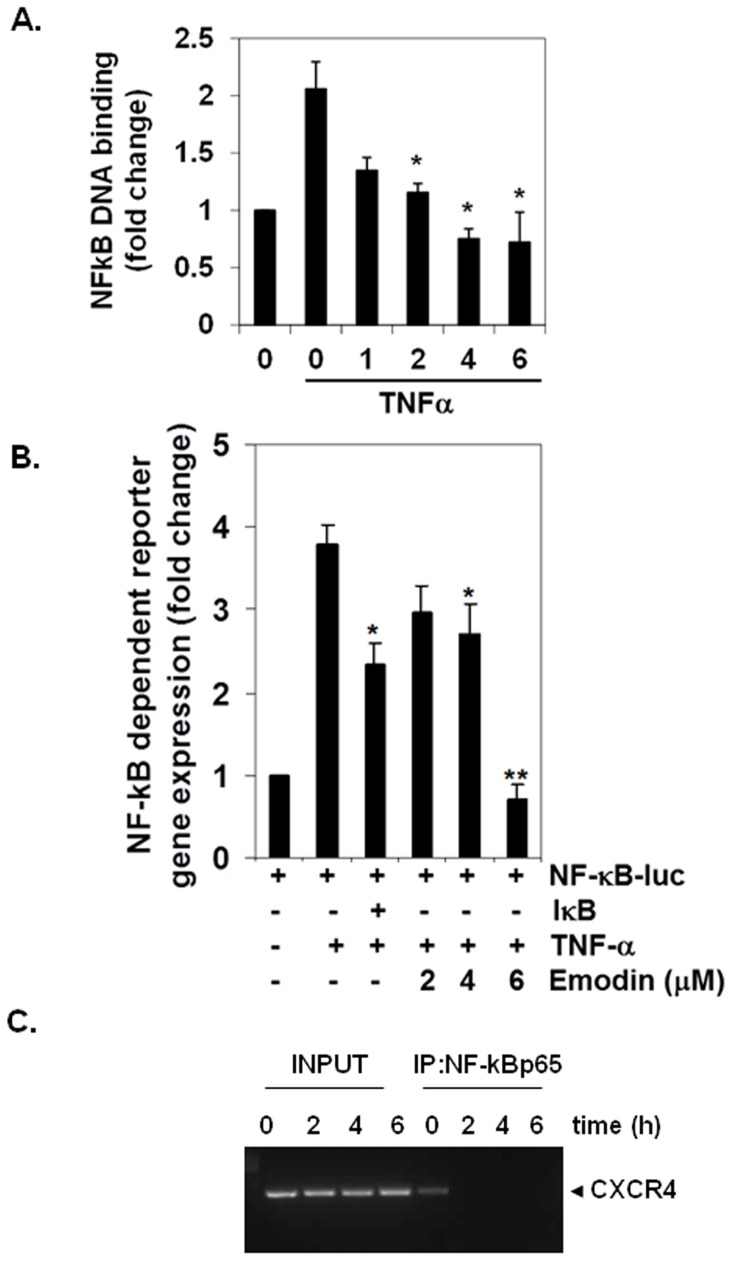
Emodin inhibits TNF-α inducible NF-κB activation in HepG2 cells. A, HepG2 cells were incubated with 50 µM emodin for the indicated time points and then stimulated with TNF-α (1 nM) for 24 h. The nuclear extracts were assayed for NF-κB activation by TransAM p65 transcription factor assay kit. Bars indicate standard error. * indicates p value <0.05. B, HepG2 cells were transiently transfected with an NF-κB luciferase plasmid and co-transfected with β-galactosidase and then treated with 50 µM emodin for the indicated time points and then stimulated with TNF-α (1 nM) for 24 h. Cell supernatants were thereafter collected and assayed for luciferase activity as described in Materials and Methods. Representative results of two independent experiments are shown. Results are expressed as fold activity over the activity of the vector control. Bars indicate standard error. * indicates p value <0.05; ** indicates p value <0.001. C, Emodin inhibits binding of NF-κB to the CXCR4 promoter. Hep3B cells were treated with 50 µM emodin for indicated time intervals and the proteins were crosslinked with DNA by formaldehyde and then subjected to ChIP assay using an anti-p65 antibody with the CXCR4 primer. Reaction products were resolved by electrophoresis.

The promoter of CXCR4 is known to contain several NF-κB binding sites [21]. Thus it is possible that emodin may exert its effect on CXCR4 by suppressing NF-κB activation. The effect of emodin on TNF-α inducible NF-κB activation in HepG2 cells was determined using DNA binding assay. We found that treatment with emodin suppressed NF-κB activation in a time-dependent manner (Fig. 3A). This result suggests that emodin may downregulate CXCR4 expression through inhibition of NF-κB activation. Because, DNA binding alone is not always associated with NF-κB dependent gene transcription [22], suggesting that additional regulatory steps are involved. Results of luciferase-based reporter assay also indicated that emodin inhibited NF-κB reporter activity significantly in a time-dependent manner in HepG2 cells (Fig. 3B).

### Emodin Inhibits Binding of NF-κB to the CXCR4 Promoter

Whether the downregulation of CXCR4 by emodin in Hep3B cells was due to suppression of NF-κB activation in vivo was examined by a ChIP assay targeting NF-κB binding in the CXCR4 promoter. We found that emodin suppressed the NF-κB binding to the CXCR4 promoter (Fig. 3C) thereby indicating that emodin inhibits CXCR4 expression by directly attenuating NF-κB binding to the CXCR4 promoter.

### Emodin Suppresses CXCL12-induced HCC Migration and Invasion

**Figure 4:**
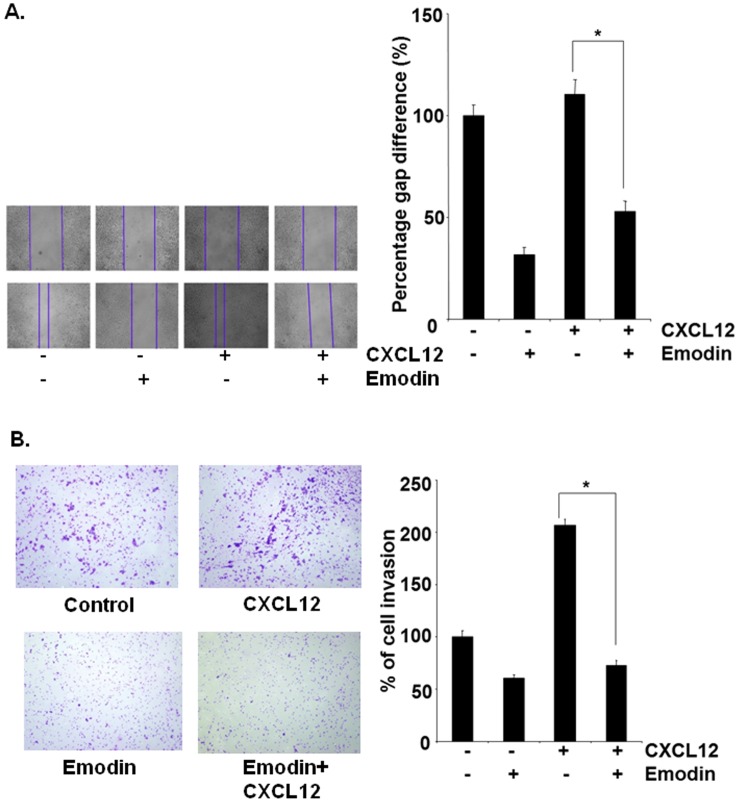
Emodin suppresses migration and invasion of HepG2 cells. A, Wound-healing assay was performed for evaluating the inhibitory effect of emodin on HepG2 cell migration. Confluent monolayers of HepG2 cells were scarred, and repair was monitored microscopically after 12 h of pre-treatment with emodin (50 µM) before being exposed to 100ng/mL CXCL12 for 24 h. Width of wound was measured at time zero and 24 h of incubation with and without emodin in the absence or presence of CXCL12. The representative photographs showed the same area at time zero and after 24 h of incubation. B, HepG2 (2×10^5^ cells) were seeded in the top-chamber of the Matrigel. After pre-incubation with or without emodin (50 µM) for 12 h, transwell chambers were then placed into the wells of a 24-well plate, in which we had added either the basal medium only or basal medium containing 100 ng/mL CXCL12 for 24 h. After incubation, they were assessed for cell invasion as described in Materials and Methods. Columns represent percentage of invaded cells; bars, S.E. *indicates p value <0.05. Representative results of two independent experiments are shown.

**Figure 5:**
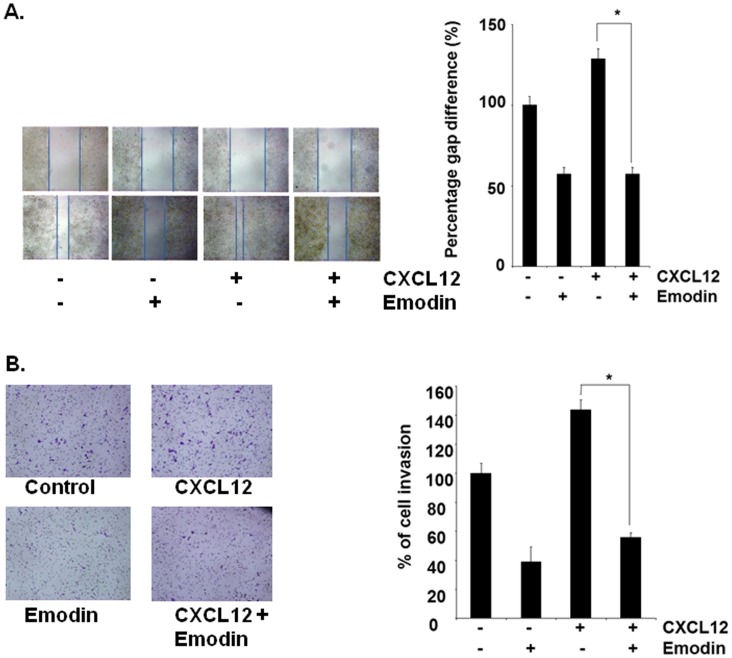
Emodin suppresses migration and invasion of Hep3B cells. A, Wound-healing assay for evaluating the inhibitory effect of emodin on cell migration. Confluent monolayers of Hep3B cells were scarred, and repair was monitored microscopically after 12 h of pre-treatment with emodin (50 µM) before being exposed to 100 ng/mL CXCL12 for 24 h. Width of wound was measured at time zero and 24 h of incubation with and without emodin in the absence or presence of CXCL12. The representative photographs of two independent experiments showed the same area at time zero and after 24 h of incubation. B, Hep3B (2×10^5^ cells) were seeded in the top-chamber of the Matrigel. After pre-incubation with or without emodin (50 µM) for 12 h, transwell chambers were then placed into the wells of a 24-well plate, in which we had added either the basal medium only or basal medium containing 100 ng/mL CXCL12 for 24 h. After incubation, the chambers were assessed for cell invasion as described in Materials and Methods. Columns represent percentage of invaded cells; bars, SE. * indicates p value <0.05. Representative results of two independent experiments are shown.

Whether downregulation of CXCR4 by emodin correlated with HCC migration was examined using an in vitro wound healing assay. We found that both HepG2 and Hep3B cells migrated faster under the influence of CXCL12 and this effect was abolished on treatment with emodin (Figs. 4A and 5A). To elucidate further the effect on emodin on CXCL12-induced cell invasion, we also found using an in vitro invasion assay, that treatment of emodin suppressed CXCL12induced invasion of both HepG2 and Hep3B cells (Figs. 4B and 5B).

### Emodin Suppresses Lung Metastasis and CXCR4 Expression in HCC Orthotopic Mice Model

**Figure 6:**
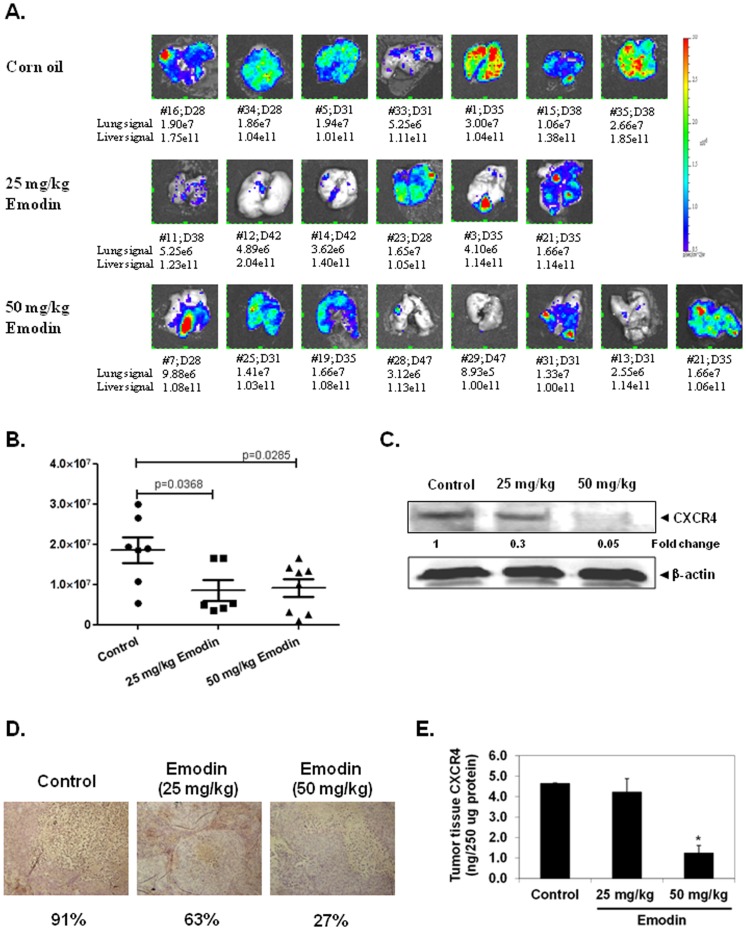
Emodin suppresses lung metastasis and CXCR4 expression in orthotopic mice model. Spontaneous metastatic model of human liver cancer cells orthotopically implanted in nude mice. Cubes of HCCLM3_Luc tumor were implanted orthotopically into the liver of female Balb/c nude mice. Mice were euthanized when the detectable liver tumor signal was >1×10^11^ p/s. A, At the time of necropsy, bioluminescent imaging was employed to monitor signals from lung metastasis. The lung biopsy was imaged to detect metastasis in the lungs. The color bar depicts the photon flux (p/s) emitted. # = mouse ID; D = day at necropsy. Lung and liver signals are in p/s unit. B, Comparison of the lung metastasis signals detected at time of necropsy for the untreated and emodin-treated groups. C, Tumor tissue extracts were prepared as described in Methods. 30 µg of protein was taken and analyzed by Western blot analysis with antibodies against CXCR4. The same blots were stripped and reprobed with β-actin antibody to show equal protein loading. D, Immunohistochemical analysis of representative section of tumor tissues stained with anti-CXCR4 antibody demonstrate strong cytoplasmic staining for CXCR4 in the control section and weak cytoplasmic staining in the emodin treated section E, Emodin decreases CXCR4 levels in tumor tissues. Tumor tissue homogenate was prepared as described in Methods. 150 µg of the tissue supernatant was taken for ELISA assay. Assay was performed according to manufacturer’s instructions using ELISA kit to determine the levels of CXCR4 in the control and treated group. Data is represented as Mean ± SE. * indicates p value <0.05.

We next investigated the ability of emodin to suppress metastasis *in vivo* using an orthotopic HCC mice model. Fig. 6A shows the ex-vivo bioluminescent images of the lungs from all tumor-bearing mice at euthanasia. All mice developed distant pulmonary metastasis from the orthotopically implanted HCCLM3_Luc tumor when the signal-to-background signal ratio of the primary tumor exceeded 1×10^11^ p/s. None of these tumors was superficially obvious or palpable. The overall metastatic signals observed in the corn oil-treated mice (1.8 x10^7^±3.22×10^6^; n = 7) were significantly higher compared to mice given 25 mg/kg emodin (8.49×10^6^±2.56×10^6^; n = 6; p = 0.0368) and 50 mg/kg emodin (9.18×10^6^±2.16×10^6^; n = 8; p = 0.0285) (Fig. 6B), demonstrating emodin treatment could significantly suppressed the development of lung metastasis.

Furthermore, we also examined whether emodin can inhibit CXCR4 expression in tumor tissues by Western blot analysis. Tissue extracts were prepared and probed with anti-CXCR4 antibody. Immunoblot analysis showed that emodin significantly suppressed the expression of CXCR4 in tumor tissue (Fig. 6C). We next investigated the effect of emodin on CXCR4 expression in mice tissues isolated from HCC orthotopic model. As shown in Fig. 6D, immunohistochemical analysis, showed strong CXCR4 expression in untreated tumor tissues and interestingly, emodin administered mice showed reduced staining thereby, indicating that emodin can suppress the expression of CXCR4 in HCC tissues. In addition, the expression level of CXCR4 in tumor tissues was also determined by ELISA assay. As shown in Fig. 6E, a significant decrease in the levels of CXCR4 was observed indicating emodin suppressed CXCR4 levels. Collectively, all these data indicate that emodin can modulate CXCR4 expression and function both *in vitro* an *in vivo* in HCC cell lines and mouse model.

## Discussion

The goal of the present study was to determine whether anti-cancer agent emodin can modulate the expression and function of CXCR4, a chemokine receptor that has been closely linked with tumor cell proliferation, invasion, and metastasis. Our results indicate for the first time that emodin downregulated the expression of CXCR4 in HCC cells, irrespective of their HER2 status. For example, emodin was found to suppress CXCR4 expression in both high HER2 overexpressing, HepG2 and low HER2 overexpressing Hep3B cells. Our results also indicated that downregulation of CXCR4 did not predominantly occur through proteolytic degradation of the receptor but rather through the downregulation of the transcript. Furthermore, emodin treatment led to the downregulation of migration and invasion induced by the ligand CXCL12 metastasis to the lungs in an orthotopic HCC mice model and CXCR4 expression in tumor tissues.

The CXCR4 chemokine receptor has been found to be overexpressed in different tumors, including HCC, breast cancer, ovarian cancer, glioma, pancreatic cancer, prostate cancer, melanoma, cervical cancer, colorectal cancer, small-cell lung carcinoma, acute myeloid leukemia, chronic lymphoblastic leukemia (CLL), B-CLL, and non-Hodgkin’s lymphoma, as compared to normal cells which show little or no CXCR4 expression [19]–[23]. Although it is still unclear, that what leads to the overexpression of CXCR4 in different tumor cells, previous reports implicate the involvement of various genetic and epigenetic factors [23]. PAX3-and PAX7-FKHR gene fusion [24], mutations in the *von Hippel Lindau* gene [25], hypoxic conditions in the tumor microenvironment [26], NF-κB [21], vascular endothelial growth factor [27], and tumor necrosis factor alpha [28] have all been found to regulate CXCR4 overexpression in different tumor cells. Recently, the epidermal growth factor receptor, c-erbB2, and its encoding gene, HER2/neu, have also been implicated in the positive regulation of CXCR4 expression at the post-transcriptional level [29], [30]. Given that CXCR4 has been linked with the metastasis, and poor survival in HCC [8], CXCR4 can be considered as an ideal molecular target for the investigation of novel therapeutic interventions for the prevention and treatment of metastatic HCC.

Our results clearly indicate that emodin suppressed CXCR4 expression in both high and low HER2 expressing HCC cells, but had minimal effect on HER2 expression in HepG2 cells.

Thereafter, we decided to investigate the various possible mechanisms by which emodin can cause downregulation of CXCR4 expression in HCC cells. One major mechanism involving the ligand-dependent downregulation of the CXCR4 receptor by lysosomal degradation has been reported previously [19]. Also, another study has indicated that E3 ubiquitin ligase atrophin-interacting protein 4 arrestin-2 complex can function on endosomes to regulate sorting of CXCR4 into the degradative pathway [20]. Moreover, Subik and coworkers recently reported that the ubiquitin E3 ligase WWP1 can negatively regulate cell migration to CXCL12 by limiting CXCR4 degradation to promote breast cancer metastasis to bone [31]. However, we found that emodin does not downregulate the CXCR4 expression entirely through this mechanism and thereby analyzed whether the inhibition of CXCR4 by this napthoquinone could possibly occur at the transcriptional level. Indeed, we found that emodin downregulated the expression of CXCR4 mRNA in HCC cells as observed by quantitative PCR analysis.

Emodin has been previously reported to downregulate NF-κB activation in various tumor cells [14]. Therefore, it is possible that downregulation of CXCR4 by emodin may occur through the suppression of NF-κB signaling, as binding site for this transcription factor has been identified in the proximal region of the CXCR4 promoter [21]. Indeed, we found that inhibition of constitutive NF-κB activation by emodin can be a causative factor for the downregulation of CXCR4 in HCC cells. Whether additional molecular mechanism(s) other than suppression of NF-κB activation are involved in downregulation of CXCR4 by emodin, cannot currently be confirmed or ruled out. Furthermore, besides CXCR4, the activation of NF-κB also regulates the expression of various inflammatory molecules including cyclooxygenase-2, matrix metallopeptidase-9, and endothelial-leukocyte adhesion molecule 1, all of which have been linked with HCC migration, invasion, and metastasis [32]. Also, since emodin can inhibit both DNA binding ability and transcriptional activation of NF-κB, as observed by us, it is possible that emodin can suppress the expression of other NF-κB regulated genes as well in HCC cells.

We further investigated the effect of emodin on CXCL12-induced migration and invasion of HCC cells. We found that pre-incubation of cells with emodin can also inhibit CXCL12-induced migration and invasion of both HepG2 and Hep3B cells. This shows the pivotal role of the CXCR4 receptor in HCC invasion and the potential of emodin to downregulate the expression or the activity of CXCR4. However, a part of our results are in agreement with a recent study by Ok *et al*. who reported that emodin indeed can inhibit the migration and invasion of prostate and lung cancer cells by downregulating the expression of CXCR4, although the potential *in vivo* effects of emodin on metastasis and CXCR4 expression was not investigated by this group [33].

Using orthotopic HCC model, we also clearly demonstrate that emodin treatment could significantly suppress the development of lung metastasis that in turn correlated with its ability to downregulate the expression of CXCR4 *in vivo* as evident by western blot analysis, immunohistochemistry and ELISA assays. The results showing that the CXCR4 expression in HCC mice tissues was suppressed by the administration of emodin is consistent with the previously reported pivotal role of CXCR4 in regulating the progression, invasion and metastasis in HCC [7]–[9]. Taken together, our findings indicate for the first time that emodin can inhibit metastasis in an orthotopic model through the modulation of CXCR4 expression in HCC. Overall, our data shows that emodin can significantly downregulate the expression of CXCR4, a key receptor involved in the crosstalk between tumor cells and its microenvironment, which contributes to its observed *in vivo* anti-metastatic effects. However, additional pre-clinical and clinical studies are needed to analyze the relevance of these observations to HCC treatment.

## Materials and Methods

### Reagents

A 50 mM solution of emodin (from Aldrich), with purity of 99%, was prepared in DMSO, stored as small aliquots at –20°C, and then diluted further in cell culture medium as needed. DMSO (0.1%) was used as vehicle control for all the *in vitro* experiments. Tris, glycine, NaCl, SDS, N-Acetyl-L-leucyl-L-leucyl-L-norleucinal (ALLN), chloroquine, and βactin antibody were purchased from Sigma-Aldrich (St. Louis, MO, USA). DMEM, fetal bovine serum (FBS), 0.4% trypan blue vital stain, antibiotic-antimycotic mixture, and HRP-conjugated secondary antibodies were obtained from Invitrogen (Carlsbad, CA, USA). Antibodies against CXCR4 and HER2 were obtained from Abcam (Cambridge, MA, USA). CXCL12 was purchased from ProSpec-Tany TechnoGene Ltd. (Rehovot, Israel).

### Cell Lines

Human hepatocellular carcinoma (HCC) cell lines HepG2 and PLC/PRF5 cells were obtained from American Type Culture Collection (Manassass, VA). HUH-7 and Hep3B cells were kindly provided by Prof. Kam Man Hui, National cancer Centre, Singapore. HepG2, Hep3B, PLC/PRF5, and HUH-7 cells were cultured in Dulbecco’s Modified Eagle Medium (DMEM) containing 1×antibiotic-antimycotic solution with 10% FBS. HCCLM3 was a kind gift of Professor Zhao-You Tang at the Liver Cancer Institute (Zhongshan Hospital, Fudan University, Shanghai) and have been described previously [34]. HCCLM3 were cultured in high glucose DMEM containing 1×antibiotic-antimycotic solution with 10% FBS.

### Western Blot Analysis

Untreated and emodin-treated HCC cells were lysed in lysis buffer (20 mM Tris (pH 7.4), 250 mM NaCl, 2 mM EDTA (pH 8.0), 0.1% Triton X-100, 0.01 mg/mL aprotinin, 0.005 mg/mL leupeptin, 0.4 mM PMSF, and 4 mM NaVO4). HCC tumor tissues (50 mg/mouse) were minced and incubated on ice for 30 min in 0.5 mL of ice-cold lysis buffer (10% NP-40; 5 M, NaCl; 1 M, HEPES; 0.1 M, EGTA; 0.5 M, EDTA; 0.1 M, PMSF; 0.2 M, sodium orthovanadate; 1 M, NaF; 2 µg/ml, aprotinin; 2µg/ml, leupeptin) and homogenized for 5 min. Lysates were then spun at 14,000 rpm for 10 min to remove insoluble material and resolved on a 10% SDS gel and western blotting was done as described previously [35].

### NF-κB Reporter Assay

HepG2 cells were plated in 96-well plates with 1×10^4^ cells per well in 10% FBS containing DMEM. After overnight incubation, cells were transfected with a NF-κB reporter plasmid linked to a luciferase gene or with the dominant-negative IκBα (IκBα-DN) plasmid, cotransfected with β-galactosidase plasmid (Promega, WI). NF-κB luciferase plasmid was obtained from Stratagene (La Jolla, CA). Transfections were done according to the manufacturer’s protocols using FuGENE® 6 obtained from Roche, (Indianapolis, IN, USA). At 24 h posttransfection, cells were treated with emodin for the indicated time points and then washed and lysed in luciferase lysis buffer (Promega, Madison, WI, USA). Luciferase activity was measured by Tecan plate reader (Durham, NC) by using a luciferase assay kit (Promega) and was normalized to β-galactosidase activity. All luciferase experiments were done in triplicate and repeated two or more times.

### NF-κB DNA-binding Activity Assay

HepG2 cells were plated in 96-well plates with 1×10^4^ cells per well in 10% FBS containing DMEM. After overnight incubation, cells were treated with emodin for the indicated time points either in presence or absence of TNF-α- and NF-κB DNA-binding activity was analyzed using the TransAM NF-κB p65 transcription factor assay kit (Active Motif, Carlsbad, CA, USA) as described previously [35].

### RNA Extraction and PCR Analysis

Total RNA isolation was performed using TRIZOL® reagent (Invitrogen) according to the manufacturer’s instructions and RT-PCR was performed as described previously [35]. For real time PCR, 1 µg of total RNA was transcribed to generate cDNA as described previously [35]. For a 50µl reaction, 10µl of product was mixed with 1× TaqMan® Universal PCR Master mix, 2.5 µl of 20× TaqMan probes for CXCR4, 2.5µl of 20× GAPDH TaqMan probe as the endogenous control. Relative gene expression was obtained after normalization with endogenous GAPDH and determination of the difference in threshold cycle (Ct) between treated and untreated cells using 2−ΔΔCt method. Primers and probes for human CXCR4, and GAPDH were purchased as Assays-on-Demand kits (Applied Biosystems).

### Chromatin Immunoprecipitation (ChIP) Assay

Hep3B cells were processed for the ChIP assay as per the protocol described previously [35]. The antibody used for the ChIP was NF-κB (p65) antibody from Santa Cruz Biotechnology (SantaCruz, CA, USA). The sequence for human CXCR4 gene promoter was follows: sense primer, 5′-ACAGAGAGACGCGTTCCTAG-3′ and antisense primer, 5′-AGCCCAGGGGACCC TGCTG-3′. The PCR products were analyzed on 2% agarose gel electrophoresis and documented.

### Wound Healing Assay

HCC cells were treated with emodin as described above. Before plating the cells, two parallel lines were drawn at the underside of the wells, to serve as fiducial marks demarcating the wound areas to be analyzed. Prior to inflicting the wound, the cells should be fully confluent. The growth medium was aspirated off and replaced by calcium-free PBS to prevent killing of the cells at the edge of the wound by exposure to high calcium concentrations before two parallel scratch wounds were made perpendicular to the marker lines with a sterile 1000-µL automated pipette tip. Thereafter, the calcium-free medium was then changed to medium with or without emodin. After incubation for 12 h with emodin, the growth medium was then changed to basal medium with CXCL12. 24 h later, the wounds were observed using bright field microscopy and multiple images were taken at areas flanking the intersections of the wound and the marker lines at the start and end of the experiment. Gap distance of the wound was measured at three different sites using Photoshop software, and the data were normalized to the average of the control. Graphs were plotted against the percentage of migration distance the cells moved before and after treatment, normalized to control.

### Invasion Assay

The in vitro invasion assay was performed using Bio-Coat Matrigel invasion assay system (BD Biosciences, San Jose, CA), as described previously [35].

### Orthotopic HCC Spontaneous Metastasis Model

All procedures involving animals were reviewed and approved by the SingHealth Animal Use and Care Committee. Five millions human HCCLM3 cells expressing firefly luciferase (HCCLM3_Luc) in 0.05 ml was injected subcutaneously into the rear flank of 10-week old female Balb/c nude mouse (Biolasco, Taiwan). When the tumor size reached approximately 1 cm^3^, tumor was harvested, cut into 1 mm^3^ pieces and surgically implanted in the liver of fresh Balb/c nude mice. Tumor growth was monitored weekly by bioluminescent imaging using the IVIS™ camera system (Xenogen, Alameda, CA, USA). Mice were randomized and treatment was started once stably increasing bioluminescence signals were observed. Mice were given 25 mg/kg or 50 mg/kg emodin in corn oil 5 days a week by intra-peritoneal injection. As control, corn oil only was given. Tumor-bearing mice were euthanized once the tumor signal reached above 1×10^11^ photons per second (p/s). For *ex vivo* imaging, 150 mg/kg D-luciferin (Xenogen) was injected into the mice intraperitoneally just before necropsy. Lungs were excised, placed into tissue culture dish and imaged for 1 min. Regions of interest in the displayed images were quantified using Living Image software (Xenogen).

### Immunohistochemistry

At the end of treatment period, HCC tumor tissue was fixed with 10% phosphate buffered formalin, processed and embedded in paraffin. Tissue sections, 5 micron size, were cut and deparafinized as described previously [36]. Sections were incubated with primary antibody antiCXCR4 at 1∶75 dilution. Negative control sections were incubated in 2% bovine serum albumin. Immunohistochemical analysis was performed according to the manufacturer’s instructions using DAKO LSAB kit. Images were taken using Olympus BX51 microscope (magnification, 20X).

### Enzyme-linked Immunosorbent Assay (ELISA)

The effect of emodin on CXCR4 levels in tumor tissue extracts was determined using CXCR4 ELISA kit (USCN Life Science Inc, USA) according to manufacturer instructions. Briefly, tumor samples were thoroughly rinsed in ice-cold PBS to remove excess blood, weighed, cut into small pieces and kept on ice. 500 µL of PBS containing protease inhibitor was added to the tissue fragments and homogenized using a tissue homogenizer on ice. The resulting tissue extract was sonicated for 30 seconds and subjected to two freeze thaw cycle to ensure complete breakdown of cells. The homogenate was centrifuged for 10 min at 10,000×g. The supernatant was taken, protein concentration was determined using Bradford reagent and assayed immediately. 150 µg of the protein was taken for ELISA assay and the sample CXCR4 concentration was determined by comparing to the standard.

### Statistical Analysis

The experiments were carried out in triplicates and repeated twice. The significance of differences between groups was evaluated by Student’s t-test and a p value of less than 0.05 was considered statistically significant.
